# Genotype of Immunologically Hot or Cold Tumors Determines the Antitumor Immune Response and Efficacy by Fully Virulent Retargeted oHSV

**DOI:** 10.3390/v13091747

**Published:** 2021-09-01

**Authors:** Tatiana Gianni, Valerio Leoni, Mara Sanapo, Federico Parenti, Daniela Bressanin, Catia Barboni, Anna Zaghini, Gabriella Campadelli-Fiume, Andrea Vannini

**Affiliations:** 1 Department of Experimental, Diagnostic and Specialty Medicine, University of Bologna, 40126 Bologna, Italy; tatiana.gianni3@unibo.it (T.G.); valerio.leoni2@unibo.it (V.L.); marasanapo91@hotmail.it (M.S.); federico.parenti5@unibo.it (F.P.); daniela.bressanin2@unibo.it (D.B.); 2 Department of Veterinary Medical Sciences, University of Bologna, 40126 Bologna, Italy; catia.barboni@unibo.it (C.B.); anna.zaghini@unibo.it (A.Z.)

**Keywords:** oncolytic virus, oncolytic herpes simplex virus, retargeting, HER2, tumor genotype, vaccination, immunotherapy, immune checkpoint inhibitors

## Abstract

We report on the efficacy of the non-attenuated HER2-retargeted oHSV named R-337 against the immunologically hot CT26-HER2 tumor, and an insight into the basis of the immune protection. Preliminarily, we conducted an RNA immune profiling and immune cell content characterization of CT26-HER2 tumor in comparison to the immunologically cold LLC1-HER2 tumor. CT26-HER2 tumor was implanted into HER2-transgenic BALB/c mice. Hallmarks of R-337 effects were the protection from primary tumor, long-term adaptive vaccination directed to both HER2 and CT26-wt cell neoantigens. The latter effect differentiated R-337 from OncoVEXGM-CSF. As to the basis of the immune protection, R-337 orchestrated several changes to the tumor immune profile, which cumulatively reversed the immunosuppression typical of this tumor (graphical abstract). Thus, *Ido1* (inhibitor of T cell anticancer immunity) levels and T regulatory cell infiltration were decreased; *Cd40* and *Cd27* co-immunostimulatory markers were increased; the IFNγ cascade was activated. Of note was the dampening of IFN-I response, which we attribute to the fact that R-337 is fully equipped with genes that contrast the host innate response. The IFN-I shut-down likely favored viral replication and the expression of the mIL-12 payload, which, in turn, boosted the antitumor response. The results call for a characterization of tumor immune markers to employ oncolytic herpesviruses more precisely.

## 1. Introduction

Our laboratory generated oncolytic herpes simplex viruses (oHSVs) detargeted from natural receptors and retargeted to cancer-specific receptors of choice [1,2,3,4,5,6,7,8,9]. These belong to the family of tumor-associated antigens (TAAs) and are targetable also thanks to their overexpression or amplification in cancer cells [10,11]. The detargeting-retargeting approach confers cancer-specificity, albeit not absolute since any given TAA exhibits a limited expression in a few off-tumor tissues [10]. It differs from the broad group of attenuation strategies, which confer cancer-selectivity and are based on deletion or mutation in virulence genes, including the γ_1_34.5 gene [12], or in genes that contrast the innate and adaptive immune responses [13,14]. The vast majority of oHSVs in clinical trials, and the approved talimogene laherparepvec (also named OncoVEXGM-CSF) were generated by the latter strategy [15,16,17]. The retargeted oHSVs carry no deletion or mutation in genes that contrast the antiviral innate and adaptive immune responses and, like wt-HSVs, are equipped to overcome the inimical host response. Once they infect the cancer cell, their replication proceeds in unattenuated fashion and is independent of defects in immune pathways typical of cancer cells. Inasmuch as the retargeted oHSVs infect specifically tumor cells and effectively spare off-tumor cells, their safety profile in mice proved to be high [1,6,18,19]. For the attenuated oHSVs, safety rests on inhibition of off-tumor infection by the innate response, and off-tumor safety. The retargeted oHSVs showed proof-of-principle efficacy upon systemic administration [18,20,21].

Our major focus has been on model tumors that express HER2 (human epithelial growth factor receptor 2) [1,2,3,21], a receptor present in a variety of indications, including a fraction of breast, ovary, stomach, lung, pancreas, and biliary tract cancers, and glioblastomas [22,23]. Despite the remarkable clinical benefits provided by anti-HER2 antibodies and inhibitors [24], the HER2-positive cancers, especially the metastatic ones, still represent unmet clinical needs. Three generations of tropism-retargeted oHSVs were produced in our laboratory [1,7,8,25]. They all carry (i) deletions in gD to detarget the HSV tropism from the natural receptors nectin1 and HVEM, (ii) the insertion of a single chain antibody (scFv) to the targeted TAA receptor in gD or in other entry glycoproteins; (iii) the most advanced ones carry a second retargeting moiety for infection of producer cells [2,5,25,26]. The third generation prototype is named R-337 and is armed with a single peptide form of murine interleukin 12 (IL-12) [25]. Repeated intratumoral administrations in LLC1 (Lewis lung carcinoma 1) tumors transgenic for human HER2 (LLC1-HER2) and implanted in C57BL/6 mice transgenic/tolerant to human HER2 (HER2-TG), protected 80% mice from primary tumors; the protection was in part immune-mediated [25] and was higher when the retargeted oHSV was armed with IL-12 [6,25]. The major modifications to the tumor microenvironment (TME) included Th1 polarization, an increase in tumor infiltrating effector cells (CD4+, DCs, CD8+, and NK cells, and their activated subpopulations), a reduction in myeloid-derived suppressor cells (MDSCs) and an increase in T regulatory cells (Tregs). The combination of R-337 with anti-PD-1 exerted a synergistic effect, even though the LLC1 tumor was completely insensitive to anti-PD-1 monotherapy. A remarkable effect of R-337 was vaccination against the LLC1-HER2 tumors, and consequently, full protection from subsequent distant challenge tumors.

Human tumors vary greatly in the mechanisms by which they evade the immune-mediated anti-tumor protection, and in immune cell composition [27,28,29,30,31,32]. On one side are immunogenic tumors (also named “immunologically hot”), characterized by T cell infiltration, Th1 cytokine expression, and drivers of immune-suppression, like immune inhibitory molecules, PD-l/PDL-1 and CTLA-4 axis, IL-10, TGF, and Tregs. On the opposite side are non-immunogenic, also defined as “immunologically cold”, or “immunologically desert” tumors, characterized by scarce T cell infiltrate, low immune activation markers, poorly expressed MHC-I, low expression of active drivers of immune suppression (immune checkpoint, Tregs), and high infiltration of MDSCs. The different immune profiles predict different prognosis for patients, result in different outcomes of immunotherapy and different sensitivity to the immune checkpoint inhibitors (ICIs) [33,34]. An intrinsic characteristic that greatly affects tumor response to immunotherapy is the mutational neoantigen load and the related response to ICIs [34,35]. Altogether, in order for murine tumor models to serve as reliable predictors, it is critical that they mirror the diversity in immune landscapes seen in human tumors [30].

The LLC1 tumor cells employed in our previous immunotherapy studies [6,25] recapitulate essentially non immunogenic tumors that do not respond to ICIs. Well characterized examples of immunogenically hot murine tumors that respond to ICIs include the CT26 and the Renca tumors, syngeneic with BALB/c mice [27,28,31].

CT26 and LLC1 tumors originated by different ways. The former was obtained by exposing mice to the N-nitroso-N-methylurea carcinogen [36]; the latter arose spontaneously. While the two tumors are similar in terms of mutational load and presence of MDSCs, and in predicted MHC-I-binding neoantigen load [31], numerous studies have set the CT26/BALB model as the most immunogenic tumor among the commonly used syngenetic murine cancers [27,31]. Hallmarks of CT26 immunogenicity are a high infiltration by leucocytes and effector immune subpopulations, a functional and highly expressed MHC class I system, high expression of both immune-activation and immune-suppression genes, high cytolytic activity (CYT) quantified as granzyme A and perforin expression, reduced in vivo tumor growth yet normal in vitro replication rate, and partial sensitivity to anti-CTLA-4 therapy [27,31,36]. LLC1 tumors stand on the opposite side of the immunogenicity spectrum, and are characterized by scarce infiltration of effector cells, low expression of immune-related genes, low MHC class I levels, rapid in vivo tumor growth, and insensitivity to ICIs [27]. Further differences between the CT26 and LLC1 tumors include the different sensitivity of the parental mouse strains to wt-HSV infection, the tissues from which they originated, the MHC haplotype and levels, the driver mutations, and the repertoire of immunodominant TAAs, etc. [27,31].

The objective of this study was to evaluate the efficacy, the modifications to the TME immune profile, and the long-term immunotherapeutic effects induced by the HER2-retargeted R-337 in the immunologically hot CT26 tumor and to obtain an insight on how R-337 induces the antitumor immune response. To this aim, we generated CT26-HER2 cells and implanted them in ad hoc generated HER2-TG BALB/c mice. Previously, we showed that the IL-12-armed HER2-retargeted oHSV exerts higher immunoprotection than its unarmed parent [6].

## 2. Materials and Methods

### 2.1. Cell Line and Virus

Human ovary SK-OV-3 cancer cells (Roswell Park Memorial Institute, Buffalo, NY, USA), CT26 colon carcinoma murine cells (ATCC, Manassas, VA, USA), and its derivative were cultured in RPMI-Glutamax (Gibco-Thermo Fisher Scientific, Waltham, MA, USA) containing 10% fetal bovine serum (FBS). RS cells were grown in DMEM (Gibco) supplemented with 5% fetal bovine serum (FBS). The CT26 cells were made transgenic for a chimeric form of HER2, carrying the transmembrane and cytoplasmic tail of the non-signaling receptor nectin1, named as “HER2-nectin”, by lentiviral transduction, as detailed [6]. Transduced cells were selected by means of puromycin and single cell clones were obtained by limiting dilution. Clones were checked for stable HER2 expression for up to 40 passages in cell culture by flow cytometry (BD C6 Accuri) with an anti-HER2 antibody (clone 9G6, Santa Cruz Biotechnology, Dallas, TX, USA). R-337 was described [25] and cultivated in SK-OV-3 cells. To measure virus growth, CT26-HER2 cells were infected at a multiplicity of 0.1 PFU/cell for 90 min at 37 °C. Unabsorbed virus was inactivated by means of acidic wash (40 mM citric acid, 10 mM KCl, 135 mM NaCl, pH 3). Replicate cultures were frozen at the indicated times (24, 48, and 72 h) after infection; the progeny was titrated in SK-OV-3 cells and scored 3 days later.

### 2.2. Mice and In Vivo Experiments

C57BL/6 mice transgenic for and tolerant to human HER2 [6] were obtained from Wayne State University (Detroit, MI, USA) through The Jackson Laboratories (Bar Harbor, ME, USA). BALB/c mice were obtained from Charles River Laboratories (Wilmington, NC, USA). Both strains and its derivatives were bred in the facility of the Department of Veterinary Medical Sciences, University of Bologna (Bologna, Italy). BALB/c-HER2 transgenic/tolerant mice were obtained by backcrossing C57BL/6-HER2 mice onto a BALB/c genetic background, for 5 generations. At each round of backcrossing, ear samples of the offspring were employed for DNA extraction (NucleoSpin Tissue, Macherey Nagel, Duren, Germany), which was used for SNPs genotyping in comparison to BALB/c WT mice (569 SNPs, Envigo, Indianapolis, IN, USA). HER2-TG mice with the highest percentage of BALB/c genetic background were employed for the following round of backcrossing. At the fifth round, the huHER2-bearing mice, which had more than 99.3% BALB/c genetic background, were considered BALB/c-HER2 mice and the mouse colony maintained by crossing huHER2-TG animals with BALB/c WT mice, without further SNPs genotyping. The animals for tumor implantation were HER2-transgenic (HER2-TG), determined by huHER2 PCR assay (The Jackson Laboratory) or BALB/c WT, where indicated. CT26-HER2 cells were implanted subcutaneously in the left flank of 7–12 week-old BALB/c-HER2 mice in 100 μL of serum-free medium, 1 × 10^6^ cells/mouse. When indicated, mice were subcutaneously engrafted with the CT26-wt cells in the right flank, in 100 μL of serum-free medium, 1 × 10^6^ cells/mouse. Tumor volumes were scored 3 times weekly by measuring the largest and the smallest diameter by means of a caliper. Tumor volume was calculated using the formula: largest diameter × (smallest diameter)^2^ × 0.5. Mice were sacrificed when tumor volumes exceed 1500–2000 mm^3^, ulceration occurred, or animals exhibited distress or pain. For virus treatment, when the tumor volumes averaged 70–100 mm^3^ (7–10 days post tumor engraftment), mice received the indicated intratumoral injections of R-337 (doses of 5 × 10^7^, 1.5 × 10^7^, or 1 × 10^7^ PFU depending on the experiment, per injection in 50 µL of PBS) or vehicle (50 µL PBS), at 3–4 day intervals. The mice that survived the primary tumor were engrafted with challenge tumors made of CT26-HER2 (left flank) or CT26-wt cells (right flank). The challenge tumors were not treated. Where indicated, mice received 6 intra peritoneal (i.p.) injections of anti-mouse CTLA-4 antibody (200 µg/mouse, clone 9H19, BioXcell, Lebanon, PA, USA), at 2–4 day intervals or vehicle (50 µL PBS).

### 2.3. Characterization of Tumor Microenvironment Infiltrating Cells

Single cell suspensions were prepared from freshly isolated CT26-HER2 tumors and spleens at sacrifice. The tumor specimens were minced and digested with collagenase (1 mg/mL) for 1 h at 37 °C. The resulting cell suspensions were passed through 70 μm cell strainer and rinsed with flow cytometry buffer (PBS+2% FBS). Spleens were processed directly by means of the cell strainer, and the red blood cells in splenic specimens were lysed by means of ACK buffer (150 mM NH_4_Cl, 10 mM NaHCO_3_, 1 mM EDTA), samples were pelleted and resuspended in flow cytometry buffer. For each sample, 2 × 10^6^ cells were blocked with α-CD16/32 Ab (clone 93, eBioscience-Thermo Fisher Scientific, Waltham, MA, USA), and then reacted with the antibodies CD4-FITC (clone GK1.5, eBioscience), CD8a-PE (clone 53–6.7, eBioscience), CD45-FITC (clone 30-F11, eBioscience), CD45-Percp-Cy7 (clone 30-F11, eBioscience), FoxP3-PE (clone 150d/e4, eBioscience), CD11b-FITC (clone M1/70, eBioscience), PD-L1-APC (clone MIH5, BD, Franklin Lakes, NJ, USA), and HER2-APC (clone 9G1D4B10, Sinobiological, Beijing, China). The data were acquired by means of BD C6 Accuri. Only the samples that provided at least 1 × 10^5^ events were included in subsequent analysis.

### 2.4. Determination of Intratumoral Cytokine and Immune Factors by RT-PCR and Expression Arrays

A few mgs of the tumor homogenates were employed for RNA purification with the NucleoSpin RNA kit (Macherey-Nagel), including the on-column DNaseI treatment. For the RT-PCR assays, 2 μg of total RNA was employed for the cDNA synthesis using the High-Capacity cDNA Reverse Transcription Kit (Applied Biosystems-Thermo Fisher Scientific, Waltham, MA, USA) in a 20 μL reaction. qRT-PCR reactions were performed in a StepOnePlus System (Applied Biosystems) using 0.5 μL of cDNA for each assay. The TaqMan probes employed for the assay were: mm00434169_m1 *(Il12a*), mm01168134_m1 (*Ifng*), mm00444662_m1 (*Cxcl11*), mm00443258_m1 (*Tnf*), mm00442837_m1 (*Gzmb*), mm00450960_m1 (*Tbet*), mm01612987_g1 (*Rpl13a*). For the expression arrays, 5 μg of total RNA was employed for the cDNA synthesis using the High-Capacity cDNA Reverse Transcription Kit, in 50 μL reaction. The assay was performed in a StepOnePlus System using 0.5 μL of cDNA for each assay of the array. The TaqMan probes employed in the arrays are listed in Table S1. The levels of expression were determined using the ΔΔCt method, normalized on the pool of *Rpl13a*, *Gapdh,* and *Gusb* housekeeping genes.

### 2.5. Detection of Splenocyte Reactivity to Cancer Cells

Freshly explanted spleens were smashed through a 70 μm cell strainer in PBS to isolate splenocytes. Red blood cells in spleens were lysed with ACK buffer, while the splenocytes were resuspended in medium (RPMI 1640 containing 10% heat inactivated FBS, 1% penicillin/streptomycin), counted and seeded in 24 well plate. Suspensions of CT26-wt and CT26-HER2 cells were treated with mitomycin 15 μg/mL (Sigma-Aldrich, St. Louis, MO, USA) for 2 h at 37 °C, then washed 3 times with fresh medium. Splenocytes (1 × 10^6^ cell/well) were incubated either with 1 × 10^5^ CT26-wt or CT26-HER2 cells in 0.5 mL medium, and cocultured for 72 h. Media were collected and the amount of secreted IFNγ was quantified by ELISA (IFN-gamma Mouse ELISA Kit, Thermo Fisher Scientific, Waltham, MA, USA).

### 2.6. Detection of Serum Antibodies to Cancer Cells and to HSV

To detect the serum antibodies to cancer cells, CT26-wt and CT26-HER2 cells were trypsinized and resuspended in flow cytometry buffer. For each sample, 0.25 × 10^6^ cells were reacted with mouse serum, diluted 1:150, in 96 well plate in ice for 1 h, rinsed with flow cytometry buffer, and finally incubated with anti-mouse APC (1:200, eBioscience). The data were acquired by means of BD C6 Accuri. To detect the serum antibodies to cancer cells, cell enzyme-linked immunosorbent assay (CELISA) was performed as described [37]. Briefly, RS cells were infected with HSV-1 (F) at 3 PFU/cell, in 96 well plate. Twenty-four hours later, they were fixed with paraformaldehyde, reacted with mouse serum diluted 1:60, or with MAb HD1 diluted 1:400, followed by anti-mouse peroxidase. Finally, peroxidase substrate o-phenylenediamine dihydrochloride (OPD; Sigma-Aldrich) was added and plates were read at 490 nm with GloMax Discover System (Promega Corporation, Madison, WI, USA).

## 3. Results

### 3.1. Generation of CT26-HER2 Cells and Their Engraftment in HER2 Transgenic/Tolerant BALB/c Mice

The BALB/c-huHER2 mouse strain was obtained by backcrossing C57BL/6-huHER2 mice [6] onto a BALB/c genetic background for five generations. At each crossing, the genome of the huHER2-bearing offspring was characterized by a panel of 569 strain typing SNPs (Envigo) to determine the percentage of BALB/c genetic background. The mice with the highest number of BALB/c genes were employed for the next round of backcrossing. At the end of the procedure, the huHER2-positive mice carried more than 99.3% BALB/c genetic background (herein BALB-HER2). The BALB-HER2 mice were bred for more than one year and maintained stable huHER2 expression.

**Figure 1:**
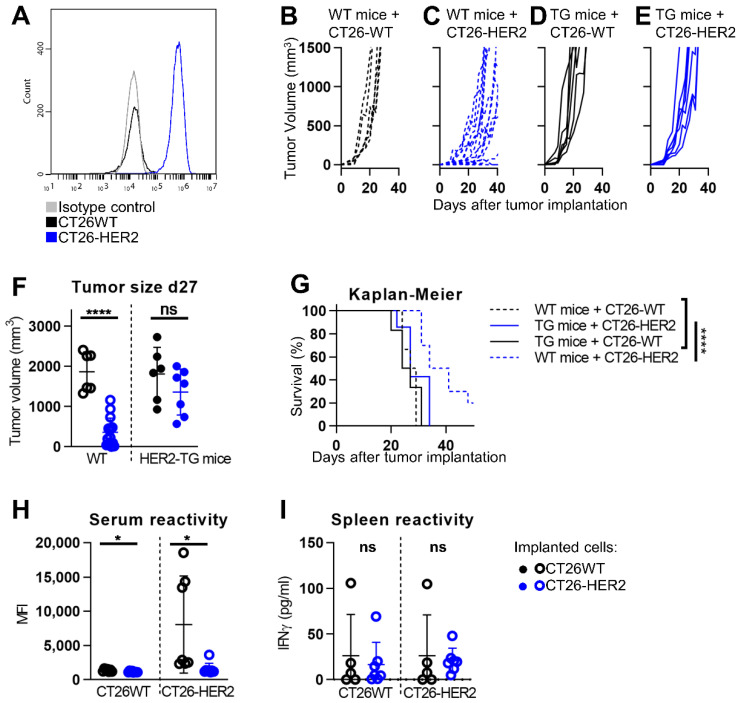
CT26-HER2 tumor development in human HER2 transgenic BALB/c mice. (**A**) Expression of HER2 in CT26 cells transgenically expressing human HER2 (CT26-HER2) determined by flow cytometry by means of anti-HER2 9G6 Mab. CT26 wild type (black), CT26-HER2 (blue), isotype control (grey). (**B–E**) Growth kinetics of CT26-HER2 (C, E) or CT26-wt (CT26) (B, D) tumors, s.c. implanted in wt- (B, C) or in HER2-transgenic/tolerant (HER2-TG) (D, E) BALB/c mice, 1 × 10^6^ cells/mouse. Tumor volume was calculated using the formula: largest diameter x (smallest diameter)^2^ × 0.5. Mice were sacrificed when tumor volumes exceed 1000–2000 mm^3^, ulceration occurred, or animals exhibited distress or pain. (**F**) Size of CT26-wt and CT26-HER2 tumors at day 27 after s.c. implantation in wt- or HER2-TG mice. (**G**) Kaplan–Meier survival curve of wt- or HER2-TG mice bearing CT26-wt or CT26-HER2 tumors. (**H**,**I**) Immune response in blood (**H**) or splenocytes (**I**) to CT26-wt or CT26-HER2 cells in spleens and blood harvested at sacrifice of wt mice. (**H**) Antibodies to CT26-wt or CT26-HER2 cells in sera harvested at sacrifice. CT26-wt and CT26-HER2 single cell preparations were reacted with mouse serum, diluted 1:150 in flow cytometry buffer (PBS + 2% FBS), in ice for 1 h, washed with flow cytometry buffer, and incubated with anti-mouse APC (1:200). Data were acquired on BD C6 Accuri. (**I**) To isolate splenocytes, spleens were smashed through a 70 µm cell strainer in PBS, red blood cells were lysed with ACK buffer, and samples were resuspended in medium (RPMI 1640 containing 10% heat inactivated FBS, 1% penicillin/streptomycin). Splenocytes (1 × 10^6^ cell/well) were incubated with 1 × 10^5^ CT26-wt or CT26-HER2 cells in 0.5 mL medium, and cocultured for 48 h. The amount of secreted IFNγ (quantified by ELISA) was a measure of the splenic anti-CT26-wt or anti-CT26-HER2 immune response. (F-I) Statistical significance was calculated by the *t*-test (**F**,**H**,**I**) or by Log-rank (Mantel-Cox) test (**G**) and expressed as * = *p*-value < 0.05; **** = *p*-value < 0.0001; ns = non-significant. Color codes: mice engrafted with CT26-wt (black) or CT26-HER2 (blue). Full circles and continuous lines, HER2-TG mice. Open circles and dotted lines, wt mice.

The murine CT26 carcinoma cells were made HER2-transgenic by lentiviral transduction, puromycin selection, and single cell cloning. The CT26-HER2 cells expressed HER2 at high level (Figure 1A) and the HER2 expression was stable for more than 40 consecutive passages, the highest number of passages tested. The CT26-HER2 cells were assayed for ability to support R-337 replication. The cells were infected at 0.1 PFU/cell, according to titer determined in the same cell line. The progeny virus yields were 1.2 × 10^6^ PFU/mL, 1 × 10^7^ PFU/mL, 1.5 × 10^7^ PFU/mL at 24, 48, and 72 h after infection. The growth kinetics of CT26-wt and CT26-HER2 tumors in wt and HER2-TG BALB/c mice is shown in Figure 1B–E. The engraftment in HER2-TG mice could not be differentiated whether the cells were wt or HER2-positive (Figure 1, compare D to E); accordingly, the volume of the CT26-wt and CT26-HER2 tumors was not significantly different at d 27 (Figure 1F, right panel). In contrast, the engraftment of the CT26-HER2 tumor in wt mice was delayed relative to that of CT26-wt tumor cells, and the volume of the two types of tumors in wt mice was highly significantly different at d 27 (Figure 1F, left panel). The differences were reflected in the Kaplan–Meier survival curve, which was statistically different for the wt mice bearing CT26-HER2 tumors and was essentially similar for the other three groups (Figure 1G). The results imply that the wt mice, but not the HER2-TG mice, delayed the growth of the HER2-positive tumor.

Analysis of the T and B immune response showed that indeed wt mice mounted an antibody response to HER2-positive, but not to CT26-wt cells (Figure 1H). Anti-HER2 splenic response was not detected in either group of mice (Figure 1I).

### 3.2. Immune Profiling of CT26-HER2 Tumors

**Figure 2:**
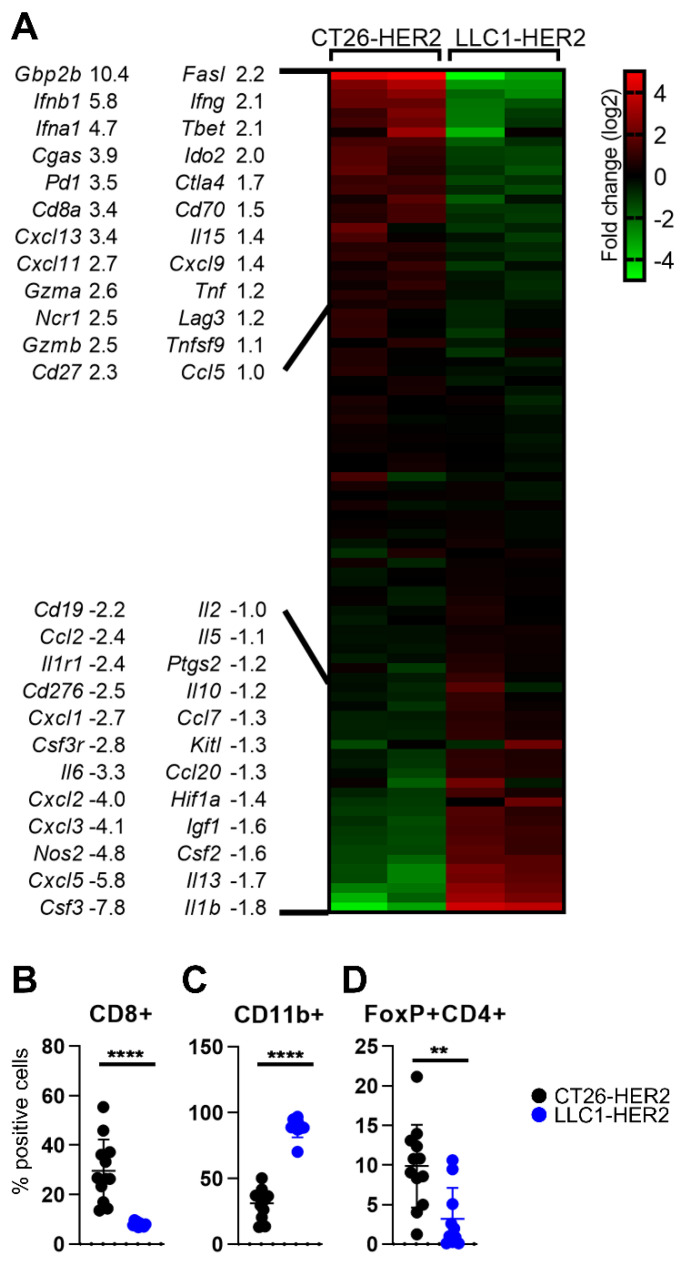
Immune profile of CT26-HER2 and LLC1-HER2 tumors (**A**) Expression array. Few milligrams of CT26-HER2 and LLC1-HER2 tumor homogenates were employed for total RNA extraction. Purified RNAs were employed for the cDNAs synthesis. cDNAs were assayed by the real-time PCR array described in Table S1. The Ct values were normalized on the *Rpl13a* house-keeping gene and, for each gene, expression levels were calculated as log2 fold change values with respect to the average ΔCt value of the gene. Differentially expressed genes (log2 fold changes>|1|) and log2 fold change values are reported. (**B**–**D**) Immune cell populations in tumors. Single cell suspensions were prepared from freshly isolated CT26-HER2 and LLC1-HER2 tumors at sacrifice. Tumors were minced in small pieces, digested with collagenase, passed through 70 μm cell strainer, and rinsed with FACS buffer. For each sample, 2 × 10^6^ cells were blocked with α-CD16/32 Ab (clone 93) and then reacted with the antibodies: CD8a-PE (clone 53-6.7), CD45-Percp-Cy7 (clone 30-F11), FoxP3-PE (clone 150d/e4), and CD11b-FITC (clone M1/70). Data were acquired on BD C6 Accuri. CD8 (CD8+ cells) and myeloid cells (CD11b+ cells) were gated on CD45+ subpopulation. Tregs (FoxP3+ CD4+) were gated on CD4+ population. (**B**–**D**) Statistical significance was calculated by the *t*-test and expressed as ** = *p* value < 0.01; **** = *p* value < 0.0001. Color codes: mice engrafted with CT26-HER2 (black) or LLC1-HER2 (blue).

In order to preliminarily define the main immune characteristics of the CT26-HER2 tumors and compare them to those of the previously employed LLC1-HER2 tumors [6,25], we performed a transcriptional analysis of 92 immune-related genes (Table S1) in CT26-HER2 and LLC1-HER2 tumor specimens (Figure 2A). In agreement with previous studies [27,31,36], we found that CT26-HER2 tumors were enriched with markers of infiltrating immune effectors, immune-activation and immune-suppression factors, interferons, and CYT. Specifically, increased *CD8a* and *Gzmb* levels denoted CD8+ T cells infiltration and activation, while higher *Ncr1* and *Gzma* were indicative of NK accumulation, since murine *Gzma* is mainly expressed by NK cells, at a difference with its human counterpart, which is expressed also by T cells [31]. CT26-HER2 specimens showed the upregulation of cytokines for T cell activation (*Il15*, *Ccl5*), of the Interferon machinery including IFN-I, -II, and IFN-stimulated genes (*Ifna1*, *Ifnb1*, *Ifng*, *Cxcl9*, *Cxcl11*, *Gbp2b*) of *cGAS* and of a global Th1 phenotype (*Tbet*). Moreover, co-stimulatory genes (*Cd27*, *Cd70*) and tumor necrosis factors (*Tnf*, *Fasl*, *Tnfsf9*) resulted in being significantly increased, along with inhibitory genes (*Pd1*, *Ctla4*, *Lag3*, *Ido2*). These results confirm and extend the paradigm that the higher expression of anti-tumor factors is balanced by increased expression of immune suppression genes, which counteract the immune activation and thus enable tumor growth [27,31].

The LLC1-HER2 tumors showed scarce infiltration by T and NK effector cells, lower expression of antitumor cytokines, and factors, but also of the above-indicated inhibitory genes. Transcriptional analysis highlighted high levels of genes that promote the infiltration by tumor-associated macrophages (TAMs) and monocytes (*Ccl2*, *Csf2*, *Ccl20*, *Ccl7*), B-cells (*Cd19*, *Il5*), granulocytes (*Csf3*, *Csf3r*, *Csf2*, *Nos2*), and, especially, tumor-associated neutrophils (TANs) (*Cxcl5*, *Cxcl3*, *Cxcl2*, *Cxcl1*). These features suggest a high infiltration by MDSCs. Higher expression of immunosuppressive cytokines capable of pro-tumoral reprogramming (*Il10*, *Il6*), immune checkpoint (*B7-H3*), hypoxia-related and non-related factors that promote tumor proliferation and metastasis (*Hif1a*, *Kitl*, *Igf1*, *ptgs2*), and pro-angiogenic factors (*Cxcl5*, *Hif1a*) were also detected. We observed the concomitant expression of anti-tumor factors (*Il1b*, *Il13*, *Ccl7*, *Il2*). Some of the transcriptional analysis results and the higher Treg infiltration were confirmed by flow cytometry (Figure 2B–D).

Globally, the poorly immunogenic LLC1-HER2 tumor appeared to grow undisturbed by the immune system, which seemed to be almost unaware of it. In contrast, the highly immunogenic CT26-HER2 tumor was recognized by the immune system and appeared to actively fight for its survival by adopting several strategies (CTLA-4, LAG3, PD1, and Tregs) that reverse the activity of the antitumor immune effectors.

### 3.3. Efficacy of R-337 Monotherapy against CT26-HER2 Primary Tumors

**Figure 3:**
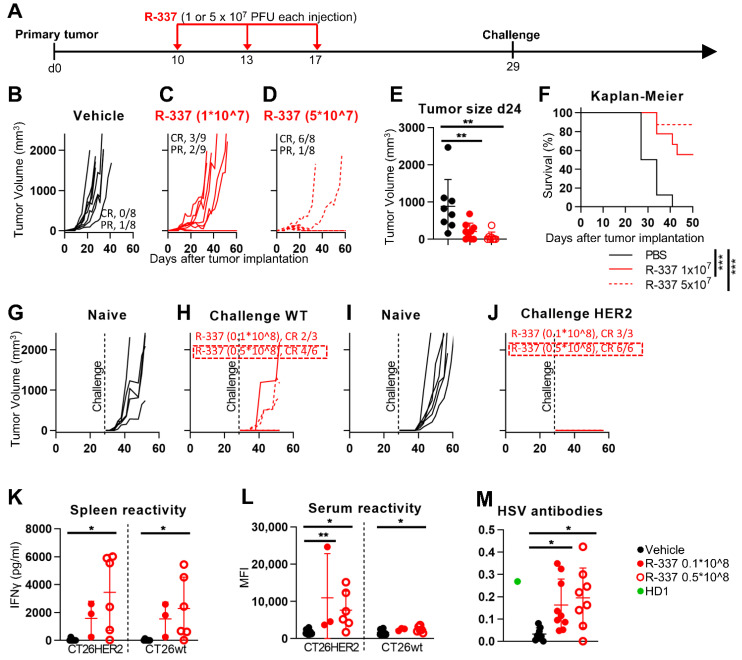
Efficacy of R-337 monotherapy on the growth of CT26-HER2 tumors. (**A**) Schedule of treatments. Six-to-eight week-old HER2-TG BALB/c mice were s.c. implanted in the left flank with 1 × 10^6^ CT26-HER2 cells in 100 μL of PBS. At d 10 after implantation, when the tumor volumes averaged 70–100 mm^3^, mice received 3 intratumoral injections of R-337, 1 × 10^7^ PFU (**C**) (R-337 low), or 5 × 10^7^ PFU (D) (R-337 high) per injection, diluted in 50 μL PBS, or vehicle (50 μL PBS), at intervals of 3–4 days. At d 29, the mice that survived the primary tumor received two contralateral challenge tumors made of CT26-wt and CT26-HER2 cells (1 × 10^6^ each, per mouse), in the right and left flanks, respectively. (**B–D**) Kinetics of tumor growth in mice treated with vehicle (**B**), R-337 low (1 × 10^7^ PFU) (**C**), or R-337 high (5 × 10^7^ PFU) (**D**). The numbers reported in each panel indicate the numbers of mice that were completely cured from tumors (complete response, CR), or which showed a delay/reduction in tumor growth (partial response, PR). The mice were scored PR when the tumor volume was <50% smaller than the mean size of the tumors in the vehicle group. (**E**) Volumes of the primary tumors at d 24 after implantation. (**F**) Kaplan–Meier survival curves of the three groups of mice. (**G–J**) Kinetics of growth of challenge tumors in naïve mice (**G**,**I**), or in the R-337 survivors’ arm (**H**,**J**). Mice received simultaneously CT26-wt (**G**,**H**) and CT26-HER2 cells (**I**,**J**). (**K–M**) Immune response in splenocytes (**K**) and sera (**L**,**M**) harvested at sacrifice from naïve mice or from the R-337 low and R-337 high arms to CT26-HER2 or CT26-wt tumors cells or to HSV1-infected cells. (**K**,**L**) Details as in legend to Figure 1. (**M**) Antibody reactivity to HSV-1 in sera harvested at sacrifice from naïve mice or from the R-337 low and R-337 high arms. RS cells were infected with HSV-1 at 3 PFU/cell for 24 h, then they were fixed with paraformaldehyde, reacted with mouse serum diluted 1:60, or with the anti-gD monoclonal antibody HD1 (green) diluted 1:400 (positive control), followed by anti-mouse peroxidase. Peroxidase substrate o-phenylenediamine dihydrochloride was added and plates were read at 490 nm as detailed [37]. (**E**,**F**,**K**–**M**) Statistical significance was calculated by means of the ANOVA test (**E**,**K**–**M**) or the Log-rank (Mantel-Cox) test (**F**) and expressed as * = *p* value < 0.05; ** = *p* value < 0.01; *** = *p* value < 0.001. Color codes: mice treated with vehicle (black), R-337 low (red, full circles and continuous lines) or R-337 high (red, open circles and dotted lines). Reactivity of Mab to HSV gD (green).

To evaluate the anti-tumor efficacy of R-337 monotherapy, the HER2-TG BALB/c mice were implanted subcutaneously (s.c.) with CT26-HER2 cells. Mice bearing well-developed tumors received three intratumoral (i.t.) injections of R337, at three to four day time intervals, as depicted in Figure 3A. Two different dosages were applied, 1 × 10^7^ PFUs and 5 × 10^7^ PFUs, respectively. Figure 3B–D shows that at the lower dosage complete response (CR) and partial response (PR) were observed in 3/9 and 2/9, respectively (33% full protection). At the higher dosage, CR and PR were observed in 6/8 and 1/8 mice, respectively (75% full protection). Determination of the tumor volumes at d 24 showed statistically significant differences were achieved with each treatment relative to vehicle treatment (Figure 3E). Such differences were reflected in the Kaplan–Meier survival curve (Figure 3F). Of note, the extent of protection was higher than that observed with the LLC1-HER2 tumors, in which three doses of 3 × 10^7^ PFUs each conferred 36% full protection. The differences may be due, in part, to different sensitivities to HSV infection exhibited by the two mouse strains. The former is known as the most resistant to HSV [38].

### 3.4. Efficacy of R-337 Monotherapy against CT26-HER2 Challenge Tumors

To ascertain whether the R-337-infected tumor cells subjected to immunogenic death plus the IL-12 adjuvant conferred long-term distant protection, i.e., vaccinated mice against the tumor, the mice that survived the primary tumor received distant challenge tumors made of CT26-HER2 and, later, of CT26-wt cells. Figure 3J shows that the growth of CT26-HER2 tumors was completely prevented. Surprisingly, the growth of CT26-wt tumors (Figure 3H) was also inhibited; 6/9 mice exhibited no tumor growth (67% protection).

The immune base of the protection from distant tumors is supported by the finding that mice developed specific T and B responses. Splenocytes taken at sacrifice and serum antibodies exhibited reactivity to CT26-HER2 and CT26-wt cells (Figure 3K,L). As expected, mice mounted an antibody response also to HSV (Figure 3M). Altogether, the efficacy results with CT26-HER2 tumors agree with our previous study on R-337-treated LLC1-HER2 tumors [25]. The remarkable difference was that in the LLC1-HER2 study, we did not observe any substantial protection from LLC1-wt challenge tumors (CR and PR in 0/4 and 2/4, respectively; 0% protection). Thus, the two model systems differ in the long-term immune response. In the current model, mice developed an immune response also towards the CT26-wt cell neoantigens.

### 3.5. R-337-Induced Modifications to Tumor Microenvironment

**Figure 4:**
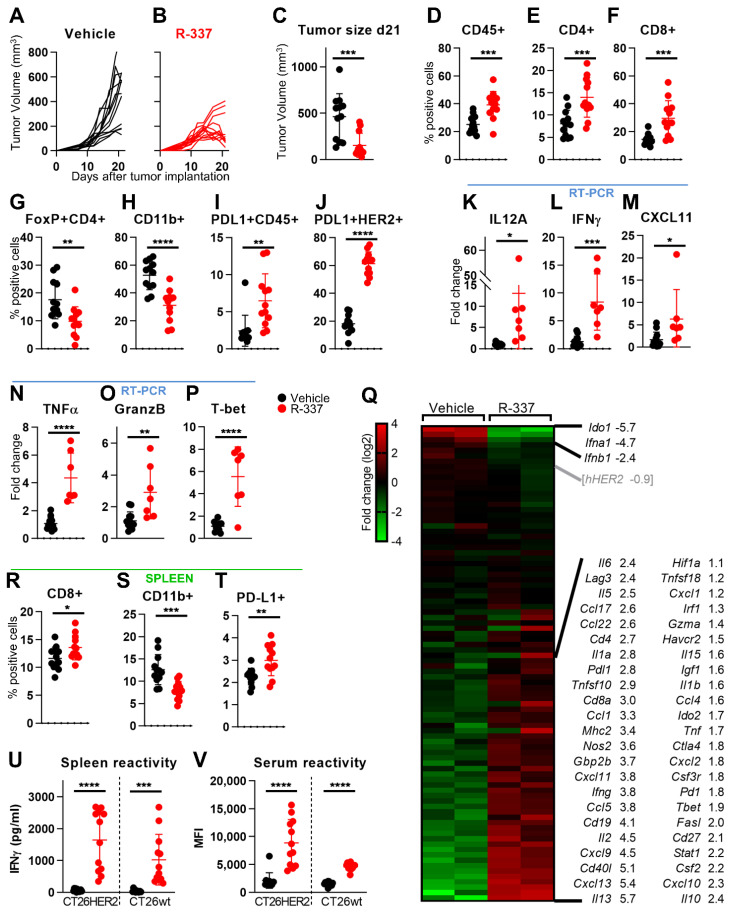
Immune modifications to TME and spleen induced by intratumoral R-337 monotherapy. (**A**,**B**) Kinetics of tumor growth in HER2-TG BALB/c mice treated with vehicle (**A**) or R-337 (**B**), according to the schedule reported in Figure 3A (3 injections of 5 × 10^7^ PFU). (**C**) Tumor volumes at d 21. (**D–H**) Immune cell populations in tumors. Samples were prepared as described in Figure 2. For each sample, 2 × 10^6^ cells were blocked with α-CD16/32 Ab (clone 93), and then reacted with the antibodies CD4-FITC (clone GK1.5), CD8a-PE (clone 53-6.7), CD45-Percp-Cy7 (clone 30-F11), FoxP3-PE (clone 150d/e4), and CD11b-FITC (clone M1/70). Data were acquired on BD C6 Accuri. CD4 (CD4+ cells), CD8 (CD8+ cells), and myeloid cells (CD11b+ cells) were gated on CD45+ subpopulation. Tregs (FoxP3+CD4+) were gated on CD4+ population. (**I**,**J**) PD-L1 expression in immune cell population or tumor cells in explanted tumors. Samples prepared as detailed above were reacted with the antibodies: CD45-Percp-Cy7 (clone 30-F11), PD-L1-APC (clone MIH5), and HER2-APC (clone 9G1D4B10). PDL1 expressed by immune cells was gated on CD45+ population, PD-L1 expressed by tumor cells was gated on HER2+ cells. (**K–P**). Expression profile of cytokines, immune-related transcription factor, and immune markers. cDNAs were prepared as detailed in legend in Figure 2. The levels of expression were determined using the ΔΔCt method, normalized on the *Rpl13a* housekeeping gene, and on the mean of the vehicle-treated group. (**Q**) Expression array. cDNAs obtained from two vehicle-treated and two R-337-treated tumors were assayed as detailed in legend in Figure 2. Differentially expressed genes (Log2 fold changes > |1|) and log2 fold change values are reported. hHER2 fold change value is under the selected threshold and is reported in grey. (**R**–**T**) Immune cell populations in spleens. Sample preparation and staining as described for tumors. (**U**,**V**) Immune response in splenocytes and sera to CT26-HER2 and CT26-wt cells was measured as detailed in legend to Figure 1. (C-P, R-V) Statistical significance was calculated by the *t*-test and expressed as * = *p* value < 0.05; ** = *p* value < 0.01; *** = *p* value < 0.001; **** = *p* value < 0.0001. Color code, mice treated with vehicle or R-337 are indicated in black or red, respectively.

The next series of experiments was designed to analyze the modifications to TME induced by R-337 infection. Mice bearing CT26-HER2 tumors were treated as in Figure 3A and were sacrificed five days after the end of the treatment, i.e., before the tumor volumes regressed too much (Figure 4A–C). Comparison of the immune cells infiltrating the untreated or R-337-treated tumors showed that the latter exhibited an overall increase in CD45-positive cells, in particular CD4+ and CD8+ (Figure 4D–F). Interestingly, a decrease was observed in the FoxP3+ CD4+ population (Figure 4G), which contains T regulatory lymphocytes, and in the CD11b+ population (Figure 4H), which includes monocytes and macrophages. PD-L1 was increased both in the lymphocyte population and in the HER2+ tumor cells (Figure 4I,J). Comparison of the cytokine profile by RT-PCR showed that R-337 induced a generalized increase in IL-12, IFNγ, CXCL11, TNFα, in effector Granzyme B, and in the T-bet transcription factor (Figure 4K–P).

For a more detailed analysis of the R-337 effects on the CT26-HER2 TME, we carried out the transcriptional analysis of 92 immune-related genes (Table S1) in specimens of virus- or vehicle-treated tumors (Figure 4Q). The expression of about half of the genes were increased in the R-337-treated tumors, suggesting a sustained TME heating. The induction matched the changes in infiltrating immune cell and cytokines described above (Figure 4D–P), and likely accounted for the decrease in tumor growth (Figure 4B). The reduction in tumor volumes was confirmed by lower human-HER2 mRNA levels (herein considered as a marker of the tumor cells) even though the variations were below the selected threshold (Figure 4Q). Most of the R-337-upregulated genes were highly expressed in the untreated tumor (Figure 2A), suggesting that the highly immunogenic tumor underwent additional immune heating. In agreement with flow cytometry results, virus-treated tumors showed higher *Cd8a* and *Cd4* markers indicative of increased infiltration by CD8+ and CD4+ subpopulations (see Figure 4E,F). *Gzma* levels were increased, hinting to active NK cells. CT26-HER2 specimens showed an increase also in the cytokines that favor T cell activation (*Cxcl13*, *Il2*, *Il15*, *Ccl5*, *Ccl4*) [39], in IFN-II and IFN-II-stimulated genes (*Ifng*, *Cxcl9*, *Cxcl10*, *Cxcl11*, *Gbp2b*, *Stat1*, *Irf1*), in co-stimulatory genes (*Cd27*, *Cd40lg*), in tumor necrosis factors (*Tnf*, *Fasl*, *Tnfsf18*, *Tnfsf10*), and in a global Th1-antitumor phenotype (*Tbet*, *MHC-II*), along with higher expression of inhibitory genes (*Pd1*, *Pdl1*, *Ctla4*, *Lag3*, *Ido2*, *Havcr2*). Increased expression of *Ifng*, *Cxcl11*, *Tnf*, *Tbet*, and *Pdl1* were in agreement with the flow cytometry and RT-PCR results (Figure 4I,J,L–N,P). Transcriptional analysis pinpointed the increase in CD4+ Th2 polarization markers (*Il5*, *Il13*), in cytokines for Treg engagement (*Ccl1*, *Ccl17*, *Ccl22*, *Ccl4*, Il6, Il10), in factors for the recruitment of monocytes/macrophages (*Csf2*, *Ccl4*), granulocytes/neutrophils (*Csf3r*, *Cxcl2*, *Cxcl1*), and B-cells (*Cd19*, *Ccl5*) [40,41,42,43], as well as in cytokines with pro- and anti-tumoral properties (*Il6*, *Il1b*, *Il1a*, *Ccl4*). The reduction in intratumoral MDSCs and Tregs (Figure 4G,H), the TME Th1 repolarization and the regression in tumor growth cumulatively indicate that the anti-cancer effects prevailed over suppressor cell responses and that these cytokines in part mediated a multipronged attack to the tumor. CT26-HER2 specimens showed a reduced expression of IFN-I, likely because R-337 is a wild type virus competent in counteracting the IFN-I cascade [25]. Interestingly, the IFN-induced *Ido1* mRNA levels were highly repressed in R-337-treated tumors. Since *Ido1* expression in CT26 and TME cells inhibits T cell anticancer immunity [44,45], its repression likely contributed to tumor clearance. Cumulatively, tumor R-337 infection subverted the tumor-tolerant TME, and the multipartite strategy adopted by immunogenic tumors to evade the immune response. The induction of inhibitory genes may well explain the increased sensitivity to checkpoint blockade seen in R-337-treated tumors.

The systemic immune response measured in spleen lymphocytes consisted of R-337-mediated increase in CD8+/CD45+ lymphocytes, decrease in CD11+ cells, and increase in PD-L1 in CD45+ lymphocytes; the modifications were similar to those seen in the tumor specimens (Figure 4R–T). Of note, the systemic immune response was further documented as splenocyte and serum antibody reactivity to both CT26-HER2 and CT26-wt cells (Figure 4U–V). The strong systemic immune response accounts for the distant protection from both CT26-HER2 and CT26-wt challenge tumors.

### 3.6. Efficacy of R-337 and Anti-CTLA-4 Combination Treatment

**Figure 5:**
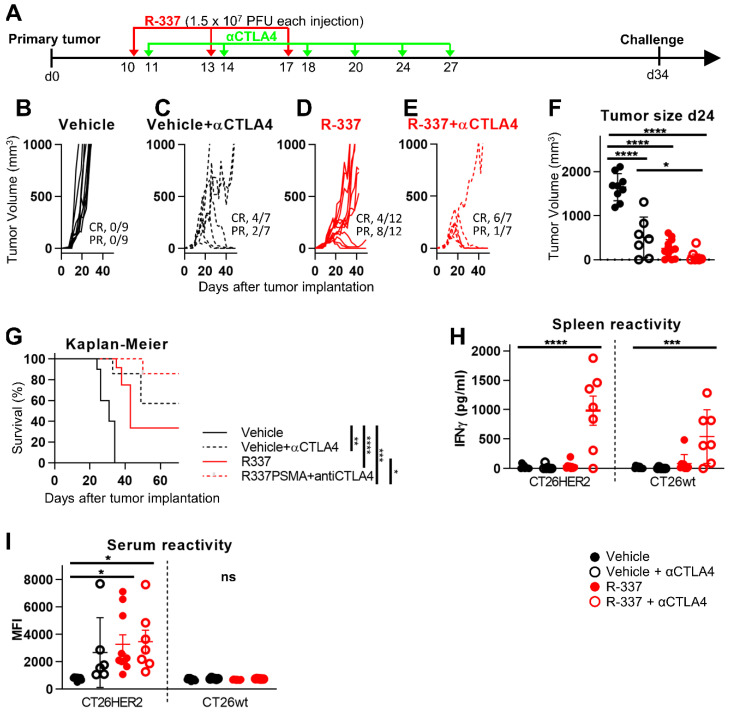
Efficacy of R-337 in combination with anti-CTLA-4 antibodies on the growth of CT26-HER2 tumors. (**A**) Schedule of the treatments. The HER2-TG BALB/c mice were implanted with CT26-HER2 cells. At d 10 after implantation, when tumors reached the average volume of 70–100 mm^3^, mice received 3 i.t. injections of R-337 plus i.p. injections of anti-CTLA-4 (abbreviated as αCTLA4), at intervals of 2–4 days. The administration schedule of R-337 and CPI treatments was according to Figure 3 and [25]. (**B–E**) Kinetics of tumor growth in mice treated with vehicle (**B**), vehicle plus αCTLA4 (**C**), R-337 alone (**D**), or R-337 plus αCTLA4 combination therapy (**E**). Numbers in panels indicate the number of mice exhibiting complete response (CR) or partial response (PR). (**F**) Volumes of the primary tumors at d 24 after implantation. (**G**) Kaplan–Meier survival curves of the four groups of mice. (**H**,**I**) Immune response in splenocytes and sera to CT26-HER2 and CT26-wt cells was measured as detailed in legend to Figure 1. H, I. Statistical significance was calculated by the ANOVA test (**F**,**H**,**I**) or the Log-rank (Mantel-Cox) test (**G**) and expressed as * = *p* value < 0.05; ** = *p* value < 0.01; *** = *p* value < 0.001; **** = *p* value < 0.0001; ns = non-significant. Color codes: mice treated with vehicle or R-337 are indicated in black or red, respectively. Full circles and continuous lines, monotherapies. Open circles and dotted lines, combination therapies.

Previously, we showed that the immune cold LLC1-HER2 tumor was highly resistant to ICI, and that it acquired sensitivity to anti-PD-1 upon treatment with R-337 [25]. Here, we asked whether R-337 could modify ICI efficacy in the CT26-HER2 tumors. The HER2-TG BALB/c mice, s.c. implanted with CT26-HER2 tumors, received three i.t. inoculations of R-337 and simultaneous i.p. treatment with anti-CTLA-4, as detailed in Figure 5A. Figure 5C shows that the growth of CT26-HER2 tumor was partially inhibited by anti-CTLA-4 monotherapy (CR in 57% of the mice). Upon three injections of 1.5 × 10^7^ PFUs each, R-337 monotherapy inhibited CT26-HER2 tumor growth (CR in 33% of the mice) (Figure 5D). The combination treatment reduced tumor growth to a higher extent than each of the monotherapies (CR in 85% of the mice; CR+PR responses in 100% of the mice) (Figure 5E). Tumor size at d 24 indicated that the reduction in tumor growth was statistically significant for each treatment and the efficacy of the combination treatment differed significantly from that of checkpoint blockade monotherapy (Figure 5F). Accordingly, the Kaplan–Meier survival curves showed highly statistically significant differences for each treatment relative to no treatment, and of the combination treatment relative to R-337 monotherapy (Figure 5G). Analysis of the splenocyte reactivity confirmed that the mice that received the combination treatment mounted a strong durable T cell response to both CT26-HER2 and CT26-wt tumor cells, i.e., to both HER2 and to the tumor neoantigens (Figure 5H). In contrast, the antibody response was addressed to CT26-HER2 cells only (Figure 5I).

## 4. Discussion

The objective of this study was two-fold; to evaluate the efficacy and the global immune response elicited by the HER2-retargeted oHSV R-337 against the CT26-HER2 tumor and to obtain an insight into the basis of immune protection. CT26 tumor is considered the prototype of immunologically hot murine tumors [27,31,36]. CT26-HER2 tumors were implanted in ad hoc generated HER2-transgenic/tolerant BALB/c mice. Previously, we reported on the immunotherapy exerted by the same virus on the immunologically cold LLC1-HER2 tumor implanted in HER2-transgenic C57BL/6 mice [25].

The initial analysis centered on RNA immune profiling and immune cell composition of the CT26-HER2 tumor. In comparison to LLC1-HER2, the CT26-HER2 tumor was characterized by an antitumoral Th1 signature and high levels of IFN-I, -II, ISGs, co-stimulatory molecules, and TNFs. This tumor-hostile environment was counterbalanced by high expression of immune-blocking factors, including different types of immune checkpoints, which likely dampened the immune response and favored tumor growth. The immune cell composition mirrored this landscape; CD8+ and NK effector cells massively infiltrated the tumor masses at the expenses of the immunosuppressive MDSCs. At the same time also, Treg cells infiltrated the tumors, and likely contributed some immune-dampening signals and an overall immunosuppressive environment (graphical abstract).

The key features of R-337 efficacy included high protection from primary tumors, protection from distant challenge tumors, partial response to anti-CTLA4 monotherapy, and increased response to combination therapy (graphical abstract). The major changes induced by R-337 to the immune profile and immune cell composition consisted in augmented Th1 polarization, i.e., in increased levels of IFN-II, ISGs, co-stimulatory molecules and TNFs, in a higher infiltration by effector CD4+ and CD8+ cells, in further depletion of MDSCs, and the concurrent increase in immune checkpoints. The Treg population was reduced (graphical abstract). These changes account for the success of the R-337 monotherapy against CT26-HER2 tumors. They differed from R-337-mediated changes to LLC1-HER2 tumors where the effector cells *de novo* populated the tumors and the infiltrating Tregs increased [25]. The protection from distant challenge CT26-HER2 tumors resulted in part from breaking of HER2 tolerance (a situation analogous to anti-HER2 protection in humans), and in part was mediated by an immune response to wt CT26 cell neoantigens. The latter protection was unexpected since OncoVEXGM-CSF failed to protect from distant CT26 challenge tumors and conferred protection only to the primary treated tumor [46]. R-337 differs from OncoVEXGM-CSF in that the latter is attenuated and rendered cancer-selective by virtue of γ_1_34.5 gene deletion. R-337 carries no attenuation or mutation/deletion in virulence and anti-innate response genes. The two viruses are armed with different cytokines, GM-CSF, and IL-12, respectively.

Current analysis indicates that the major traits of the immune protection elicited by R-337 in CT26-HER2 tumors consisted in the reversal of the immune suppressive phenotype and essentially restored the tumor immunogenicity, as follows.

(i) The amount of infiltrating T regulatory cells (Tregs) was low in the immunologically cold LLC1-HER2 tumor and high in the immunogenic CT26-HER2 tumor, where they likely contribute to dampen the tumor immunogenicity. While the amount of infiltrating Tregs increased in R-337-treated LLC1-HER2 tumors [25], the amount of Tregs decreased in R-337-treated CT26-HER2 tumors. Such a decrease likely contributed to the virus-induced reversal of the CT26-HER2 immune suppressive phenotype.

(ii) Several immunosuppressive mechanisms, including high checkpoints expression, negatively regulate CT26-HER2 immune response. The strategy deployed by R-337 to revert such actively immunosuppressive microenvironment consisted of the increase in co-stimulatory factors (e.g., *Cd40l* and *Cd27*), activation of the IFN-II signaling cascade (documented here as increase in IFNγ, in ISGs, and Th1 polarization), and the decrease in *Ido1* expression, a key ISG involved in immune escape.

(iii) The tumor genotype impacted on the effects of combination therapies. Thus, in the immunologically cold LLC1-HER2 tumor, the checkpoint molecules were expressed at low levels, and the tumor was intrinsically resistant to checkpoint blockade; R-337 resulted in sensitization to checkpoint inhibition [25]. In contrast, in the immunologically hot CT26-HER2 tumor, the checkpoint molecules and effectors, including CTLA-4 and *Ido1*, were at high levels; the tumor responded to checkpoint blockade. R-337 treatment resulted in an overall increase in the ICI efficacy; the response to the combination therapy appeared to be additive.

Of note was the antiviral innate response. IFNα and β (but not IFNγ) were dramatically decreased in R-337-treated CT26-HER2 tumors relative to untreated tumors. This was likely the consequence of the fact that R-337 is non-attenuated and puts in place the mechanisms typical of wt-HSV to dampen the antiviral innate response. Consequently, the tumor sustained the virus replication, as well as the expression of the transgenic mIL-12 payload. In turn, the high mIL-12 expression likely contributed to the Th1 antitumor phenotype [47].

The impact of the tumor genotype on the efficacy of oncolytic HSVs was reported in independent observations, for example in the mitogen-activated protein kinase kinase –protein kinase R (MEK-PKR) pathway during the infection with Δγ_1_34.5 oHSVs [48]. Cumulatively, current and previous findings call for a thorough characterization of tumor molecular markers to evaluate the extent to which the murine tumors are predictive of the diversity of human tumor immune landscapes, and to employ oHSVs more precisely in the therapy of cancers.

## 5. Patents

Campadelli G.; Menotti L. Herpes Simplex Virus (HSV) with modified tropism, uses and process of preparation thereof. WO2009144755 (divisional patent EP2700405), 4 April 2018

Campadelli G.; Gatta V. *Retargeted herpesvirus with a glycoprotein H fusion*. WO201612849, 18 August 2016

Campadelli G.; Petrovic B. *Herpesvirus With Modified Glycoprotein B*. WO2017211941, 14 December 2017

Leoni V.; Campadelli G. *Herpesvirus With Modified Glycoprotein D.* WO2017211944, 14 December 2017

Nicosia A.; Campadelli G. Herpesvirus With Modified Glycoprotein H For Propagation In A Cell. WO2017211945, 14 December 2017

## Supplementary Materials

The following are available online at https://www.mdpi.com/article/10.3390/v13091747/s1, Table S1: list of genes and Taqman probes employed in the transcriptional analysis.
